# Malposition of Central Venous Catheter into Coronary Sinus throughout the Persistent Left Superior Vena Cava and Other Complications Related to Catheterization

**DOI:** 10.3390/diagnostics14101038

**Published:** 2024-05-17

**Authors:** Barbara Syska, Anna S. Veer, Patrycja S. Matusik, Jarosław D. Jarczewski, Katarzyna Krzanowska, Tadeusz J. Popiela

**Affiliations:** 1 Students’ Scientific Group, Department of Diagnostic Imaging, Jagiellonian University Medical College, 31-008 Cracow, Poland; barbara.syska@student.uj.edu.pl (B.S.); anna.veer@student.uj.edu.pl (A.S.V.); 2 Department of Diagnostic Imaging, University Hospital, 30-688 Cracow, Poland; jaroslaw.dawid.jarczewski@gmail.com (J.D.J.); msjpopie@cyf-kr.edu.pl (T.J.P.); 3 Chair of Radiology, Faculty of Medicine, Jagiellonian University Medical College, 31-008 Cracow, Poland; 4 Department of Nephrology, Jagiellonian University Medical College, 31-008 Cracow, Poland; kasiajanda@op.pl

**Keywords:** central venous catheter, persistent left superior vena cava, imaging techniques

## Abstract

This case concentrates on the persistent left superior vena cava (PLSVC), a rare vascular anomaly which contributes to central venous catheter (CVC) misplacement. A 72-year-old woman with renal insufficiency presented to the hospital with recurrent bleeding from her permanent CVC device placed in the right common jugular vein. An initial attempt to replace the device was unsuccessful, necessitating the placement of a secondary catheter in the left jugular vein. Shortly after the procedure, the patient developed swelling of the face and neck. Further diagnostic imaging, including a chest radiograph and computed tomography (CT), revealed CVC misplacement in the PLSVC and coronary sinus, thrombosis of the common jugular vein, and a posterior mediastinal hematoma. Conservative therapy of the mediastinal hematoma was implemented and proved effective in this case. A temporary CVC was inserted into the left femoral vein. Two months later, the catheter underwent further dysfunction and a decision was made to place a long-term permanent CVC via the right femoral vein. The patient is currently awaiting an arteriovenous fistula for dialysis use. This case emphasizes the importance of radiological techniques for CVC procedural placement, as well as the detection of congenital abnormalities. Providers regularly placing CVCs should have an in-depth knowledge of the possible complications and potential anatomical variations, especially as seen in high-risk patients.

The central venous catheter (CVC), an indwelling instrument that is placed into the large vein above the heart, enables venous access and benefits a wide range of patients in a variety of clinical settings including but not limited to emergency departments, intensive care units, radiologic settings, or long-term medication management among others [1]. Indications for CVCs are widely varied, but common uses include the administration of intravenous fluids, medications, blood products, parenteral nutrition, vasoactive medications, hemodialysis, and hemodynamic monitoring [1]. The primary benefit of CVCs is facilitating easy and quick access to the patient’s bloodstream, significantly reducing the number of needle injections into the patient [1]. This reduction can help prevent needle-induced sores from intravenous therapies and improve overall patient comfort. However, CVCs may be associated with various complications, including CVC misplacement, malfunction, infection, thrombosis, catheter fragmentation during removal, and fibrin sleeve formation [2,3].

The aim of this report is to present a case of 72-year-old woman with end-stage renal failure due to glomerulonephritis. The patient was admitted to the hospital due to recurrent bleeding from a permanent dialysis catheter placed in the right common jugular vein, possibly caused by mechanical damage thus necessitating replacement. After an unsuccessful attempt to replace the catheter, a secondary catheter was placed in the left jugular vein. Immediately after catheterization, the patient, previously respiratory and circulatory stable, developed swelling of the neck and face. A chest X-ray revealed an abnormal CVC position (Figure 1a). A computed tomography (CT) study confirmed that the CVC followed a non-anatomical route and was seen on the left side, in the persistent left-sided superior vena cava (PLSVC), and entering in the coronary sinus (Figure 1b–g).

A small pericardial effusion was detected (Figure 2a). Additionally, the imaging shows a hematoma in the posterior mediastinum and common jugular vein thrombosis (Figure 2b,c), which are likely complications of the CVC insertion. The size of the hematoma, which was visible from the foramen magnum to the hiatus of the diaphragm, measured at 53 × 28 × 165 mm. Conservative therapy of the mediastinal hematoma was implemented and was effective in this case.

Due to anemia, the patient received two units of red blood cells. Consultations with a cardiologist, cardiac surgeon, and invasive cardiologist led to the recommendation to remove the dialysis catheter inserted in the left jugular vein. Therefore, the permanent hemodialysis catheter was removed, and a temporary CVC was implanted into the left femoral vein. Dialysis was performed on the temporary catheter without complications. Follow-up imaging showed visible regression of the hematoma and no fluid accumulation in the pericardial sac. Additionally, stenosis of the superior mesenteric artery around the head of the pancreas was discovered (Figure 2d).

One month later, the temporary CVC implanted into the left femoral vein developed an extensive hematoma in the soft tissues of the thigh, necessitating surgery. Surgical intervention included ligation of the bleeding arterial vessel and evacuation of the hematoma. The temporary CVC implanted in the left femoral vein was removed. After an angiology consultation, it was decided to insert a long-term permanent hemodialysis catheter into the right femoral vein. The patient is currently awaiting the creation of an arteriovenous fistula for dialysis.

Misplacement is one of the most common complications associated with CVCs, and is defined as the tip of the catheter not lying in the so-called “ideal position” [4]. The type of catheter misplacement defines its severity, with the most common (and less serious complication) being misplacement of the tip itself, and the most serious and even more rare complication being extravascular placement [4]. Catheter misplacement depends on several factors such as the site of insertion, the technique used, and the patient’s body positioning [4]. Incorrect catheter placement can lead to premature CVC failure due to vein or catheter thrombosis. The types of catheter misplacements (intravascular and extravascular) and their associated complications are depicted in Figure 3 [2,3,4,5].

The mechanisms of CVC misplacement appear to be multifactorial. Scientific research shows that difficult body habitus, such as obesity or large breasts, can promote tip migration and increase the risk of mispositioning [6]. In female and obese patients, the migration of CVCs placed in the subclavian veins is more common. When addressing the most common reasons for misplacement, one must consider that misplaced CVCs have been reported in almost every possible anatomical position, including the arterial system, mediastinum, pleura, pericardium, etc. [5]. Therefore, when assessing the different reasons for misplacement, it needs to be examined from all the major access points that are used for CVCs. Mispositioning of the CVC carries a high risk when crossing to contralateral subclavian vein or ascending internal jugular vein. Insertion into the femoral vein carries the highest risk of arterial puncture [5].

Certain congenital and acquired abnormalities predispose patients to catheter misplacement [6]. Acquired abnormalities are far more common than congenital abnormalities. Acquired obstruction of the central veins can be classified into two types as follows: those due to factors which lie external to the vein and factors which are internal, caused by the vein itself or the contents of the vein [6]. Most external factors are caused by compression due to malignancy (lung cancer, breast cancer, lymphoma, or germ cell tumors), whilst most internal factors are caused by thrombosis or stenosis. The insertion and presence of a CVC can lead to damage of the vessel wall, predisposing the patients to thrombosis [7]. Factors increasing the risk of thrombosis include recent surgery, pregnancy, and diabetes.

Most congenital anatomical abnormalities are asymptomatic and may only become apparent after CVC placement through imaging. A congenital variation that bears clinical significance is PLSVC, a persistent remnant of a vessel present alongside the normal right-sided superior vena cava (SVC) in early embryological development but which normally disappears later in development amongst healthy individuals [4]. The different types of PLSVC are depicted in Figure 4. Serious complications such as shock, cardiac arrest, angina, dangerous arrythmias, and tamponade have been reported during catheterization in adults with PLSVC; however, these are rare complications.

CVC placement techniques vary but share a common goal, which is placing the catheter tip in the available central vein, located outside the pericardial sac, in a parallel position to avoid any angulation towards the vein itself or the heart [1]. The most common placement locations are the SVC, inferior vena cava (IVC), or right atrium. Typically, the optimal location is the lower third of the SVC and the upper third of the right atrium [8]. Generally, insertion begins with a central vein (i.e., internal jugular, subclavian, or femoral), and the catheter is advanced until its terminal lumen reaches its target location [1]. Modern-day CVC placement utilizes a variety of assist techniques and devices, such as ultrasounds, real-time X-ray, or electrocardiogram (ECG) guidance to help practitioners achieve better visualization [9,10]. The ECG-guided technique allows the physician to correct the CVC tip position in real-time by observing the P-wave morphology. An increase in the P-wave height is noted when the CVC is introduced into the RA, as the intracavitary electrode is then near the sinoatrial node. If the catheter tip is further advanced deep into the right atrium, the P-wave height declines. A critical skill necessary for accurate placement is a comprehensive understanding of both physiological and variant anatomy. Imaging studies (typically beginning with X-rays) are used to confirm proper placement.

In the case presented, malposition was preventable. Using fluoroscopy during the procedure would have shown that the guidewire was traveling to the left of the spine rather than the right. This finding could suggest one of the three following scenarios: PLSVC, potential insertion into the carotid artery and descending aorta, or extra-luminal placement of the device into the mediastinum. Therefore, it is recommended that these types of procedures be performed under fluoroscopic guidance. As with most medical procedures, the level of experience of the physician reduces the risk of complications. Therefore, theoretical–practical training (theoretical class, training on mannequins and ultrasound) should lead to better procedural outcomes and reduce complications. Additionally, to reduce the probability of CVC-related infections and thrombosis, CVCs should be removed as soon as they are no longer needed.

Clinicians who routinely place CVCs should be well versed in the possible complications of the procedure, particularly in high-risk patients. It is essential to employ suitable implant techniques such as fluoroscopy and avoid blind placement methods. Properly checking the catheter tip placement and assessing for complications are critical for an early and suitable diagnosis, which is essential for better quality of treatment and patient outcomes.

## Figures and Tables

**Figure 1:**
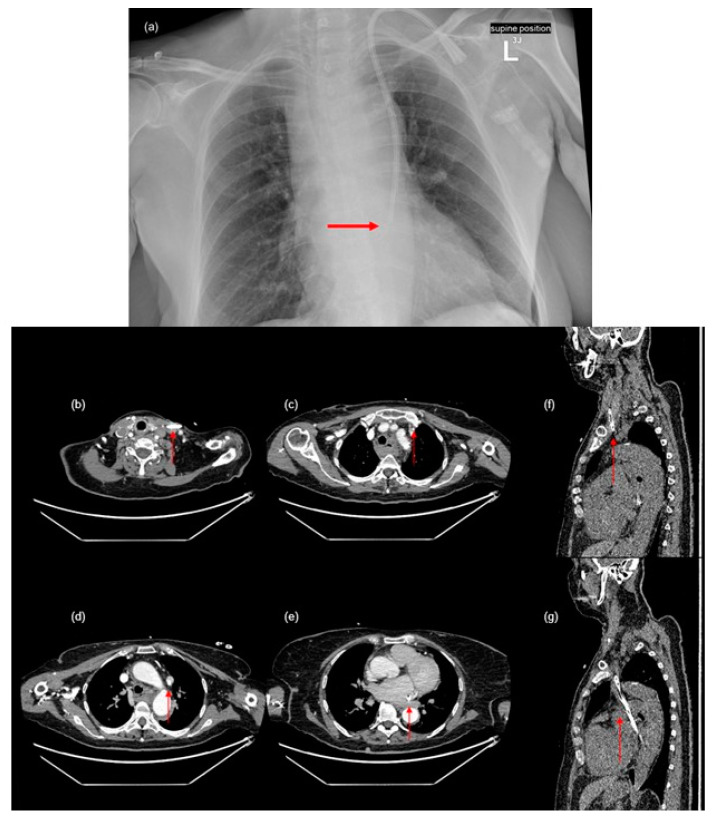
Posterior to anterior chest X-ray with a central venous catheter inserted into the persistent left superior vena cava (arrow in (**a**)). Persistent left superior vena cava in computed tomography ((**b**–**g**)—arrows indicate the path that the catheter passed).

**Figure 2:**
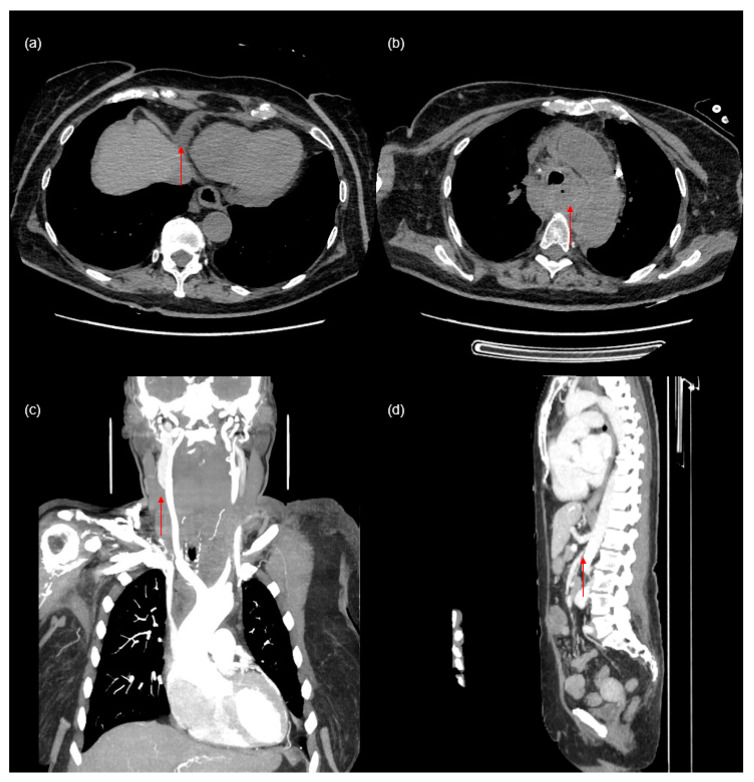
Complications following central venous catheter insertion and additional findings observed in this patient ((**a**)—pericardial effusion (arrow), (**b**)—mediastinal hematoma (arrow), (**c**)—common jugular vein thrombosis (arrow), (**d**)—stenosis of the superior mesenteric artery (arrow)).

**Figure 3:**
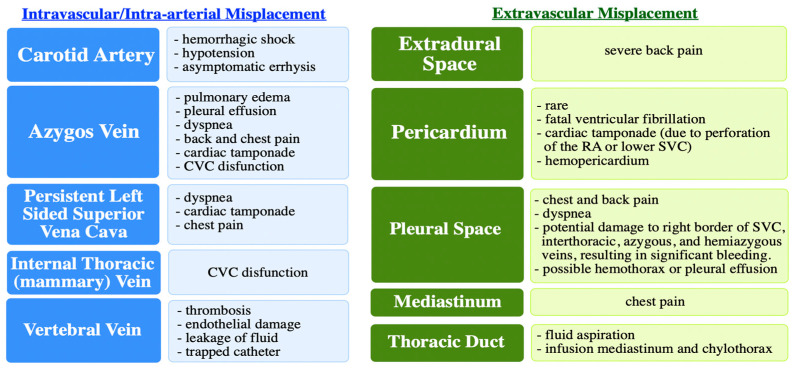
Types of catheter misplacements and their associated complications.

**Figure 4:**
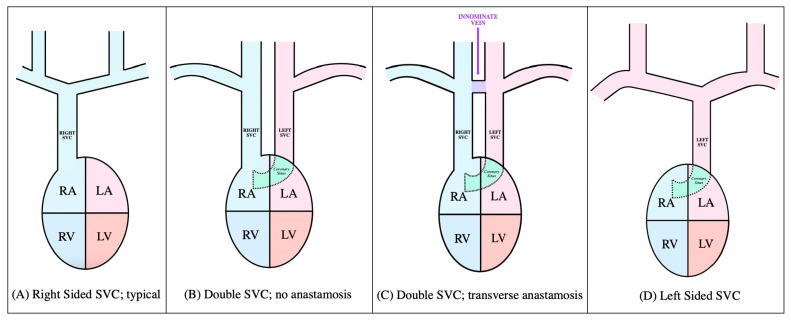
Visualized anatomical variations of persistent left superior vena cava. Abbreviations: LA—left atrium; LV—left ventricle; RA—right atrium; RV—right ventricle; SVC—superior vena cava.

## Data Availability

All relevant data are within the manuscript.
